# Regulation of Antiviral Immune Response by *N*^6^-Methyladenosine of mRNA

**DOI:** 10.3389/fmicb.2021.789605

**Published:** 2021-12-16

**Authors:** Baoxin Zhao, Weijie Wang, Yan Zhao, Hongxiu Qiao, Zhiyun Gao, Xia Chuai

**Affiliations:** ^1^Department of Pathogen Biology, Hebei Medical University, Shijiazhuang, China; ^2^Institute of Medical and Health Science, Hebei Medical University, Shijiazhuang, China

**Keywords:** *N*^6^-methyladenosine modification, viral infection, immune recognition, innate immunity, adaptive immunity

## Abstract

Host innate and adaptive immune responses play a vital role in clearing infected viruses. Meanwhile, viruses also evolve a series of mechanisms to weaken the host immune responses and evade immune defense. Recently, *N*^6^-methyladenosine (m^6^A), the most prevalent mRNA modification, has been revealed to regulate multiple steps of RNA metabolism, such as mRNA splicing, localization, stabilization, and translation, thus participating in many biological phenomena, including viral infection. In the process of virus–host interaction, the m^6^A modification that presents on the virus RNA impedes capture by the pattern recognition receptors, and the m^6^A modification appearing on the host immune-related molecules regulate interferon response, immune cell differentiation, inflammatory cytokine production, and other immune responses induced by viral infection. This review summarizes the research advances about the regulatory role of m^6^A modification in the innate and adaptive immune responses during viral infections.

## Introduction

The discovery of modifications residing in DNA and histone proteins has proposed epigenetics, which provides a new perspective on regulation of gene expression and many other important biological processes. Besides this, there are more than 170 covalent modifications in the other layer of the central dogma, RNA, predominantly in tRNA and rRNA (Boccaletto et al., 2018). Those RNA modifications, such as *N*^6^-methyladenosine (m^6^A), *N*^1^-methyladenosine (m^1^A), 5-methylcytidine (m^5^C), pseudouridine (Ψ), 2′*O*-methylation (2′OMe), 7-methylguanosine (m^7^G), and *N*^6^, 2′*O*-methyladenosine (m^6^A_m_), are critical for RNA metabolism, function, and localization, thus becoming a research hot spot (Nachtergaele and He, 2018).

Currently, emerging research indicates that m^6^A, as the ubiquitous modification in internal mRNA, is dynamically regulated by the functional interplay among m^6^A methyltransferases, demethylases, and reader proteins. It is generally believed that the “write-in” of a methyl group to the *N*^6^ position of adenosine is catalyzed by the *S*-adenosyl-L-methionine (SAM)-dependent multisubunit methyltransferase complex composed of METTL3, METTL14, and other accessory components. The m^6^A modification specifically occurs in fractional mRNA and in the consensus sequence, DRACH (D = A, G, or U; R = G or A; H = A, C or U) (Fu et al., 2014). m^6^A codes are interpreted through being bound by the particular m^6^A RNA-binding proteins, such as the YTH domain-containing proteins (YTHDC1-2, YTHDF1-3) (Wang et al., 2015). In addition, the RNA structure can be destabilized due to the weaker base pair interactions between m^6^A and U; thus, heterogeneous nuclear ribonucleoprotein (hnRNP) may be recruited to bind to the hidden RNA-binding sites (Liu et al., 2017). Therefore, m^6^A readers are used to characterize the mRNA-binding proteins whose affinity to mRNA can be influenced by the presence of m^6^A and/or m^6^A-induced RNA structure changes (Shi et al., 2019). These m^6^A readers execute the function of m^6^A in multiple processes of mRNA fate, such as splicing, nuclear export, cap-independent translation, and decay. The oxidative demethylation of m^6^A is proved to be carried out by the demethylases ALKBH5 and FTO, known as erasers, which confer the reversibility of m^6^A modification in the life cycle of mRNA (Zaccara et al., 2019). Furthermore, m^6^A modification heavily influences a variety of physiological and pathological events, such as embryonic development, cell differentiation, viral infection, and tumorigenesis by fine-tuning RNA biology (Bi et al., 2019).

Numerous studies show that viral infection can induce host m^6^A machinery rearrangement; meanwhile, m^6^A-associated proteins positively or negatively regulate the viral replication cycle and pathogenesis by changing the m^6^A modification status of viral RNA reciprocally (Yang et al., 2019). By transcriptome-wide mapping of m^6^A sites and manipulation of writers, erasers, or readers to perturb m^6^A, it is reported that the decoration of m^6^A in influenza A virus (IAV) genomic RNA or mRNA increased hemagglutinin expression (Courtney et al., 2017), whereas Zika virus (ZIKV) replication was inhibited by m^6^A modification (Lichinchi et al., 2016b). As to hepatitis B virus (HBV), m^6^A that distributed at the 5′ epsilon stem loop was required for efficient reverse transcription of pregenomic RNA (pgRNA), whereas m^6^A at the 3′ epsilon stem loop resulted in destabilization of all HBV transcripts, including mRNA and pgRNA (Imam et al., 2018). However, there are some conflicting opinions about how m^6^A modifications influence the replication of human immunodeficiency virus-1 (HIV-1) (Kennedy et al., 2016; Lichinchi et al., 2016a; Tirumuru et al., 2016). The appearance, location, and function of m^6^A modification in diverse viral RNA are summarized in detail in previous reviews (Kennedy et al., 2017; Wu et al., 2019). The outcomes of viral infections depend on not only the magnitude of virus amplification or their cytocidal effects, but also the host immune status to a large extent.

It is well characterized that innate and adaptive immune responses are invoked in succession upon a virus invading. As the host’s first line of defense against viruses, pattern recognition receptors (PRR) are critical in the recognition of conserved pathogen-associated molecular patterns (PAMPs) and launching a series of protective immune responses rapidly (Schlee and Hartmann, 2016). PRR, such as Toll-like receptors (TLR), the RIG-I-like receptor family (RLR), and the NOD-like receptor family (NLR), capture viral RNA specifically and signal through the adaptor myeloid differentiation primary response protein 88 (MyD88) or mitochondrial antiviral signaling protein (MAVS). Upon sensing viral RNA, macrophages produce a large amount of cytokines, for instance, interleukin-1β (IL-1β), IL-6, tumor necrosis factor (TNF), and interferon (IFN), eliciting inflammatory responses, building an antiviral state to block virus reproduction, and enhancing the phagocytosis or cytotoxicity effects of neutrophils and natural killer (NK) cells (Chen et al., 2017; McFadden et al., 2017). Subsequently, host cellular and humoral immune responses are often activated by antigen presenting cells (APC) to eliminate viruses. As a critical subset of CD4^+^ T cells, helper T lymphocytes (Th) function in orchestrating antiviral responses by producing cytokines, including IFN-γ, IL-2, IL-4, and IL-5 (Zhu, 2018). Regulatory T cells (Treg), as a group of immunosuppressive cells, participate in regulation of infection or inflammatory responses to minimize immune pathogenesis in infectious conditions (Rakebrandt et al., 2016). It is conceivable that, except for acting on viral RNA directly, the m^6^A modification likewise has remarkable regulatory control on the immune system and other host reactions, which gives rise to either strengthen or weaken antiviral effects. In this review, we outline the recent advances in the field about the regulation of m^6^A modification in the antiviral-related immune processes mentioned above, highlighting the innate immunity in response to viral infection.

## m^6^A Modification in Non-Self RNA Recognition

An intrinsic feature of PRR is the ability to discriminate between exogenous and host RNA, which is essential for clearance of viruses while ensuring dormancy of autoimmune responses. It is proved that RNA possessing 5′-triphosphate, double-strand, local folded, or other signatures are all recognized as non-self by PRR (Schlee and Hartmann, 2016). Given that m^6^A modifications are naturally found in most cellular mRNA, early views believed that, like the DNA restriction-modification system in bacteria, it served as a mark for immune sensors to distinguish self from non-self RNA (Sitaraman, 2016). However, the increasing discovery of m^6^A in almost all kinds of viruses demonstrates that m^6^A incorporation into viral RNA may be an approach whereby viruses imitate the host RNA to evade recognition by RLR and TLR, just like 2′OMe, another form of viral RNA modification (Ringeard et al., 2019).

### Retinoic Acid-Induced Gene I

In the presence of K63-linked polyubiquitin, RIG-I can be activated by binding with exogenous RNA and then undergo conformational change and recruit MAVS to activate the IFN transcription factors (Malik and Zhou, 2020). However, previous research illustrates that *in vitro* synthesized RNA containing m^6^A modifications binds RIG-I poorly and could not trigger RIG-I conformational conversion or induce innate immunity (Durbin et al., 2016). Similar phenomena also occurred on circular RNA (circRNA) or short interfering RNA (siRNA), and YTHDF2 binding to the m^6^A modified RNA may account for the decreased immunogenicity (Chen et al., 2019; Imaeda et al., 2019). Until recently, the role of m^6^A modification in virus immune evasion has been deciphered. According to the result of a human metapneumovirus (HMPV) infection model, m^6^A-ablated HMPV was more likely to be trapped by RIG-I but not melanoma differentiation-associated gene-5 (MDA5) and facilitated RIG-I conformational change and oligomerization. The authors conclude that the m^6^A modification inhibits type I IFN production through protecting the viral RNA from being recognized by RIG-I both *in vitro* and *in vivo* (Lu et al., 2020). Since then, several studies have been published that show m^6^A modifications on different viruses, such as HBV, HCV, HIV-1, MeV, SeV, vesicular stomatitis virus (VSV), and severe acute respiratory syndrome coronavirus-2 (SARS-CoV-2), have the same effect on the process of RIG-I recognition (Kim et al., 2020b; Chen et al., 2021; Li et al., 2021; Lu et al., 2021; Qiu et al., 2021). These studies also detail that m^6^A-modified viral RNA recruited YTHDF2 and YTHDF3, and these reader proteins sequestered the viral RNA from RIG-I sensing (Kim et al., 2020b; Lu et al., 2021). m^6^A modification could reduce the local double-stranded structure of viral RNA, which is the critical signature to be recognized by RIG-I (Qiu et al., 2021).

### Toll-Like Receptors and Other RNA Sensors

There are some similar findings in other RNA sensors, such as TLR, protein kinase R (PKR), and IFN-stimulated gene 20 (ISG20). One of the studies finds that substitution of A with m^6^A blocke the activity of RNA to activate dendritic cells (DC) *in vitro* through signaling of TLR3, TLR7, and TLR8 (Kariko et al., 2005). PKR can specifically detect highly structured viral RNA to restrain virus multiplication and is found to be activated by the less modified noncoding RNA in *NIPBL* mutated lymphoblastoid cells (Yuen et al., 2016; Bou-Nader et al., 2019). Therefore, it is very likely that m^6^A modification is involved in viral RNA sensing by PKR molecules. However, a completely contrary role of m^6^A modification was unveiled in HBV RNA. Using YTHDF2 as an intermediate, m^6^A-modified HBV RNA can be selectively recognized and degraded by ISG20 through its 3′-5′ exonuclease activity (Imam et al., 2020). Together, it still needs more systematic and in-depth research to elucidate the versatile roles and mechanisms of viral RNA m^6^A modification in innate immune recognition.

Nucleoside-modified mRNA vaccines, which not only express viral antigens stably, but also avoid being recognized and degraded by the host immune system due to the depressed immunogenicity, provide new ways for the prevention of infectious diseases. Indeed, other types of nucleoside-modified mRNA vaccines have been successfully developed against certain viruses, such as IAV, ZIKV, HIV, and SARS-CoV-2 (Pardi et al., 2017, 2018; Richner et al., 2017; Cohen, 2020), and the recently approved mRNA vaccines for emergency use authorization by FDA developed by Pfizer and Moderna are demonstrated to be very potent in stimulating strong humoral and cellular immune responses (Anderson et al., 2020; Dooling et al., 2020). Based on the findings mentioned above, it is hopeful to design mRNA vaccines by incorporating m^6^A modifications into virus mRNA.

## m^6^A Modification in Innate Immune Response

The innate immune system is the host’s inherent first line of defense against viruses. Studies reveal that many aspects of the innate immune response, such as expression of IFN and ISG, inflammatory response, macrophage and DC maturation are all tightly controlled by m^6^A modification as a consequence to either improve the antiviral effects efficiently or weaken the immune response to prevent immunopathological damage.

### Interferon Response

IFNs are a class of principal cytokines that can restrict virus amplification and spread. Binding to cell membrane receptors, IFNs activate the Janus kinase (JAK)-signal transducer and activator of transcription (STAT) pathway, leading to the transcription of a whole repertoire of antiviral ISG. To avoid the deleterious outcomes induced by excessive IFN response, strategies that the host evolves to fine-tune IFN production are equally important. m^6^A modification is linked to negative regulation of IFN-β production in normal human dermal fibroblasts triggered by human cytomegalovirus (HCMV) or dsDNA (Rubio et al., 2018). Herein, the slower biogenesis and faster decay of IFNB mRNA were involved in the underlying mechanism. Alternatively, m^6^A might deposit onto nascent IFNB mRNA co-transcriptionally, and the initiation or elongation of transcription might be obstructed by the m^6^A group. This finding is verified and extended in other similar research in which HCMV infection of primary human foreskin fibroblasts and murine CMV (MCMV) infection of mice were exploited (Winkler et al., 2019). The negative regulatory role of m^6^A on IFNB production was directly identified by comparing the expression level of putative m^6^A site-mutated with wild-type IFNB constructs. Despite the discovery that the stability of IFNB mRNA was increased when the m^6^A sites were mutated, the role of m^6^A on transcription in the earlier period was not considered in this study.

Earlier studies observe that several ISG transcripts translate effectively in the presence of RNA-binding proteins, including G3BP stress granule assembly factor 1 (G3BP1), G3BP2, and cytoplasmic activation/proliferation-associated protein-1 (CAPRIN1) (Bidet et al., 2014; Li et al., 2015). Furthermore, the interacting sites in mRNA and determinants that affect the binding of these three stress granule proteins were explored with the development of proteomics. Two proteomic studies tried to explain how m^6^A modification impacted on mRNA-protein interactions in which m^6^A modification repelled binding of G3BP1, G3BP2, or CAPRIN1 to the mRNA, and these three proteins were, therefore, proposed to be m^6^A antireaders (Arguello et al., 2017; Edupuganti et al., 2017). Hence, it can be predicted that the expression of certain ISGs could be negatively modulated by m^6^A modification as well. Given that host m^6^A-associated machinery are induced almost immediately when viral infection takes place, m^6^A modification may act as a suppressive signal to downregulate the magnitude of IFN response and restrict cytotoxicity; on the other hand, this mechanism can be hijacked by viruses to facilitate their replication.

Contradictorily, some studies report that the enhancement of IFN response is also attributed to m^6^A modification. It was indicated that, after herpes simplex virus-1 (HSV-1) infection, m^6^A modification of cyclic GMP-AMP synthase (*CGAS*), gamma-interferon-inducible protein 16 (*IFI16*), and stimulator of interferon gene (*STING*) mRNA in RAW264.7 cells led to their cytoplasm localization and expression of these transcripts, suggesting that m^6^A modification was crucial to drive type I IFN production (Wang L. et al., 2019). m^6^A modification was also found to expedite IFN production in another study by Cao and colleagues (Zheng et al., 2017). DEAD-box (DDX) helicase family member DDX46 recruited ALKBH5 *via* its DEAD helicase domain to demethylate m^6^A modified *MAVS*, TNF receptor-associated factor 3 (*Traf3*), and *Traf6* mRNA in RAW264.7 cells infected with VSV. The resultant demethylation of these three mRNAs reduced their nuclear exportation and translation into proteins responsible for IFN production, which demonstrated a positive role of m^6^A modification in IFN response. Contrarily, another DDX helicase family member DDX5, could enhance the formation of the METTL3–METTL14 complex, which methylates *p65* and *IKK*γ mRNA in the nuclear. The increased methylation of these transcripts results in accelerated degradation and negatively regulates IFN-β and IL-6 production after VSV infection (Xu et al., 2021). A recent study found that WTAP maintains the protein abundance of IRF3 and IFNAR1 by improving *IRF3* translation efficiency and *IFNAR1* mRNA stability *via* m^6^A modification (Ge et al., 2021). Coincidentally, another study revealed that m^6^A modification promotes the translation of certain ISGs during the IFN response, thus augmenting the antiviral innate immunity functions (McFadden et al., 2021).

Phosphatase and tensin homolog (PTEN), as an innate immune regulator, promotes dephosphorylation of interferon regulatory factor 3 (IRF3) at the Ser97 site with a corresponding facilitation of IRF3 nuclear import and IFN production. HBV could increase m^6^A modification of PTEN mRNA and contribute to its instability in host cells by which HBV evaded the attack from the immune system (Kim et al., 2020a). The forkhead box protein O3 (FOXO3) is a repressive transcription factor that diminishes IFN-γ production and antiviral activity. In RAW264.7 cells infected with VSV, the m^6^A reader protein YTHDF3 potentiated FOXO3 translation, and the latter downregulated ISG expression (Zhang et al., 2019). It is interesting that YTHDF3 bound to the initiation region of *FOXO3* mRNA independently of METTL3-installed m^6^A. However, the authors did not analyze the m^6^A sites on *FOXO3* mRNA or the influences of synonymous point mutation. It is still unclear whether m^6^A modification is really involved in the binding of YTHDF3 to FOXO3 mRNA. Taken together, it is clear that the biological significance of m^6^A modification for the IFN response is complex and remains to be further investigated.

### Macrophage Polarization and Dendritic Cells Activation

Classical or M1 macrophages are characterized by ingestion and digestion of cells infected with viruses and proinflammatory activity. The polarization of M1 macrophages rely on transcription factors, including STAT1 and IFN regulatory factor 5 (IRF5) although STAT6 and peroxisome proliferation-activated receptor-γ (PPAR-γ) are required for differentiation of the alternatively activated M2 macrophages that orchestrate immunoregulation, fibrous tissue repair, and restrain the duration of inflammatory response (Alisjahbana et al., 2020). It seems to be contradictory about the role of m^6^A modification in macrophage polarization in the following two studies. Through methylated RNA immunoprecipitation, *STAT1* mRNA was identified to be m^6^A modified at its 3′-untranslated region (UTR) in murine bone marrow-derived macrophages (BMDMs) (Liu et al., 2019c), and the m^6^A methylation markedly inhibited *STAT1* mRNA decay and gave rise to a constant protein translation, underlying M1 BMDMs phenotypic maturation. However, another study found that m^6^A modification resulted in decreased mRNA stability of *STAT1* and *PPAR-γ via* YTHDF2, thereby impeding both M1 and M2 macrophage polarization (Gu et al., 2020). Further analysis of the role of reader proteins that bind to these m^6^A sites would resolve this contradiction.

During activation in response to viral infection, DCs express high levels of membrane costimulatory molecules, such as CD40, CD80, CD86, and Toll/IL-1 receptor homologous region domain-containing adaptor protein (Tirap) for initiating the adaptive immune response efficiently. Research focusing on regulation of DC maturation indicates that m^6^A upregulates the expression of CD40, CD80, and Tirap to prime T lymphocytes (Wang H. et al., 2019). The m^6^A modifications in these three mRNA were recognized by YTHDF1, and subsequently, the translation was strengthened.

### Inflammatory Cytokines Production

Inflammatory responses, which are featured by local recruitment of considerable leukocytes and cytokines, are destined for suppression of infection processes. Uncontrolled inflammatory response intensity and duration, such as cytokine storm, may lead to severe immunopathological damage to the host (Cao, 2020). TLR-mediated nuclear factor kappa B (NF-κB), mitogen-activated protein kinase (MAPK), and other signaling pathways are the targets of epigenetic regulation of the inflammatory response (Yasmin et al., 2015). For instance, METTL3 facilitates activation of NF-κB and MAPK pathways in human dental pulp cells and chondrocytes (Feng et al., 2018; Liu et al., 2019b), and a completely opposite biological activity of METTL3 is found in THP-1 macrophages, in which overexpression of METTL3 significantly restrained NF-κB phosphorylation and nuclear translocation (Wang J. et al., 2019). YTHDF2 is suggested to participate in the destabilization of *MAPK* mRNA of RAW264.7 macrophages or *IL11* mRNA of hepatocellular carcinoma cells, thus reducing IL-1β, IL-6, IL-12, and TNF-α production and relieving inflammation dramatically (Hou et al., 2019; Yu et al., 2019).

In these studies, it is not compelling to draw conclusions about the regulatory role of m^6^A modification only by evaluating the effects of perturbing METTL3 or YTHDF2 on the expression of inflammation-related genes, and additional mapping of the m^6^A distribution in the transcripts of these genes is required to clarify how gene expression or the RNA process is impacted by the m^6^A modifications more convincingly. Together, these divergent findings indicate the complicated regulation role of m^6^A modification in the inflammatory response, depending on the diverse cell lines or cellular components, and a comprehensive understanding about how inflammatory response against viruses are controlled by m^6^A remains to be further studied.

### Other Innate Immune-Related Molecules

Right open reading frame kinase 3 (RIOK3) is a protein serine/threonine kinase that can phosphorylate MDA5 and maintain MDA5 at an inactive state (Oshiumi et al., 2016). Cold-inducible RNA binding protein (CIRBP) is induced under cellular stresses and can stabilize specific mRNA and facilitate their translation (Liao et al., 2017). In the context of infection by *Flaviviridae*, *RIOK3* methylation, and *CIRBP* demethylation took place, and the changed m^6^A status promoted translation of *RIOK3* and alternative splicing of *CIRBP*, respectively, all benefiting *Flaviviridae* infection consequently (Gokhale et al., 2020).

## m^6^A Modification in Adaptive Immune Response

Except the regulatory role for innate immunity, m^6^A modification was also discovered to be correlated with adaptive immune responses, for example, T lymphocyte proliferation and differentiation, DC migration to lymph nodes, and antigen presentation.

### T Lymphocyte Proliferation and Differentiation

In the process of naive T cell differentiation into Th1 and Th17 cells, IL-7/STAT5 pathway activation is pivotal. Using conditional *METTL3* knockout mice, it was observed that m^6^A deposition in the suppressor of cytokine signaling (SOCS) family *SOCS1*, *SOCS3*, and *CISH* mRNA accelerated their decay (Li et al., 2017). Consequently, the suppression to the IL-7/STAT5 signal pathway was removed, and this led to reprogramming of naive T lymphocytes. Subsequent research by Li and colleagues found that the immunosuppression function of Treg arose from IL-2/STAT5 pathway activation, and m^6^A indirectly modulated this pathway through SOCS as in Th cell differentiation (Tong et al., 2018). Another subset of T cells, follicular helper T cells (Tfh) are essential for initiating germinal center formation and activating B lymphocytes. An inducible co-stimulator (ICOS) is a signaling molecule that is indispensable for Tfh cell development. Glyceraldehyde-3-phosphate dehydrogenase (GAPDH) was identified as a key target downstream of the E3 ligase VHL-hypoxia-inducible factor 1α (HIF-1α) signaling pathway to regulate the development of Tfh. It is reported that GAPDH could promote m^6^A modification on *ICOS* mRNA to reduce protein expression, thereby inhibiting the early development of Tfh (Zhu et al., 2019).

### Migration and Antigen Presentation of Dendritic Cells

CCR7 chemokine receptor stimulation promotes the movement of DCs to draining lymph nodes rapidly for antigen presenting to T cells and the priming of adaptive immune responses (Bretou et al., 2017). Excessive DC migration and accumulation are related to variable inflammatory disorders; therefore, timely termination of DC migration is the key to orchestrating immune homeostasis. A long noncoding RNA *lnc-Dpf3* was identified as a feedback regulator for CCR7-induced DC migration (Liu et al., 2019a). CCR7 stimulation upregulated the level of *lnc-Dpf3* concurrently *via* m^6^A demethylation to prevent YTHDF2-mediated degradation, and *lnc-Dpf3* directly bound to HIF-1α and abrogated transcription of the lactate dehydrogenase A (*Ldha*). As a result, *lnc-Dpf3* inhibited glycolytic metabolism and migratory capacity of DC.

As the most powerful APC, the antigen processing and presenting of DCs can also be fine-tuned by m^6^A modification. To be specific, mRNA of lysosomal cathepsins, including CTSA, CTSB, CTSD, and CTSH, are m^6^A modified in DCs, and the expression of these proteases was reinforced by YTHDF1 (Han et al., 2019). More degradation of tumor neoantigens by these lysosomal proteases resulted in less antigen presentation, leading to the escape of tumor cells from immune surveillance. Whether the virus utilizes this immune “ignorance” caused by YTHDF1 to avoid recognition by the immune system deserves further verification. This implicates YTHDF1 as a potential therapeutic target in anticancer or antiviral immunotherapy.

## m^6^A Modification and Antiviral-Related Components

### Metabolite of Host Cells

Host cell metabolism, which encompasses metabolite availability and energy generation, can be used to shape the course of immune events and to affect the environment of viral survival. The m^6^A level on α-ketoglutarate dehydrogenase (*OGDH*) mRNA was initially increased due to the impaired enzymatic activity of ALKBH5 in RAW264.7 cells after VSV infection (Liu et al., 2019d). The m^6^A modification promotes *OGDH* transcript degradation through YTHDF2, and decreased OGDH protein expression metabolically suppresses production of itaconate, which is exploited for virus replication. This study shows the importance of m^6^A modification in the metabolomic response to viral infection.

### Immunome of Host Cells

Recent years have witnessed technological breakthroughs, such as methylated RNA immunoprecipitation sequencing (MeRIP-seq) and m^6^A individual nucleotide resolution crosslinking and immunoprecipitation (miCLIP), which made it possible to profile the m^6^A landscape at the transcriptome level (Grozhik et al., 2017; Ovcharenko and Rentmeister, 2018).

By MeRIP-seq, 56 transcripts were identified as constitutively m^6^A modified in MT4 cells upon HIV-1 infection, and the most represented categories were viral gene expression and multiorganism metabolic process (Lichinchi et al., 2016a). In fact, 19 of these genes were known to be linked to HIV replication, such as *EIF3M*, *TRAF2*, and *HNRNPK*. However, in Jurkat and primary CD4^+^ T cells, the uniquely m^6^A modified genes upon HIV-1 infection enriched in functional clusters, such as metabolism, immune system process, multicellular organismal process, and development (Tirumuru et al., 2016). Researchers subsequently found that the binding of HIV-1 envelope glycoprotein gp120 to the CD4 receptor molecule is required for the upregulation of the m^6^A modification level in recipient cells (Tirumuru and Wu, 2019). There are some similar studies focusing on host m^6^A methylome changes upon ZIKV, respiratory syncytial virus (RSV), Kaposi’s sarcoma-associated herpesvirus (KSHV), and *Flaviviridae* infections (Lichinchi et al., 2016b; Hesser et al., 2018; Tan et al., 2018; Fu et al., 2019; Xue et al., 2019).

According to the results of these studies, it is certain that viral infection can rewrite the host cell methylome, and these newly gained or lost modifications often simultaneously occur at sets of genes that are enriched in confined pathways related to viral infection even if various statistical models for m^6^A peak calling or GO analysis algorithms were applied. Considering that genes whose expression is highly regulated often contain abundant m^6^A sites in their mRNA (Gokhale et al., 2020), these modular alterations might be an effective means for modulating immune related gene expression programs to promote or restrict viral infection. It is necessary to carry out deeper studies for verifying whether and how these genes or signaling pathways are regulated by m^6^A modification.

## Who Leads the Alteration of m^6^A Modification?

It merits expanding research to determine how virus–host interactions drive the changed methylome or, in other words, the changed m^6^A machinery in the infected cells. Recently, several enlightening studies shed some light on the mechanisms. It is demonstrated that Epstein–Barr virus nuclear antigen 3C (EBNA3C) upregulates METTL14 expression depending on activation of the *METTL14* promoter and stabilizes METTL14 protein (Lang et al., 2019). As a result, the increased METTL14 level facilitates EBV proliferation and self-renewal of host cells. Investigations show that the interaction between METTL3 and enterovirus 71 (EV71) nonstructural protein 2C or 3D may contribute to the cytoplasm localization of METTL3 in rhabdomyosarcoma (RD) cells (Hao et al., 2019; Yao et al., 2020). In addition, the viral protein 2A that harbors a nuclear localization signal could compete with METTL3 for nuclear importing protein karyopherin, and this partially explains the redistribution of METTL3 after EV71 infection. Siddiqui and colleagues found that HBx, an HBV-encoded regulatory protein, could interact with m^6^A methyltransferases and guide them to the HBV minichromosome and host PTEN chromosomal locus to achieve cotranscriptional m^6^A modification (Kim and Siddiqui, 2021). In HepG2 cells, *Flaviviridae* infection-activated innate immune and endoplasmic reticulum stress controlled the alteration of *RIOK3* and *CIRBP* m^6^A conditions, respectively (Gokhale et al., 2020). The protease encoded by HIV-1 could cleave m^6^A reader protein YTHDF3, which incorporates into HIV-1 viral particles, antagonizing the limitation role of YTHDF3 on viral production and infectivity (Jurczyszak et al., 2020). Similarly, the 2A protease of enterovirus antagonizes the induction of ISGs in infected cells by cleaving m^6^A readers YTHDF1-3 (Kastan et al., 2021). These studies indeed illustrate the complex link between the viruses and m^6^A modification machineries.

## Conclusion

**FIGURE 1:**
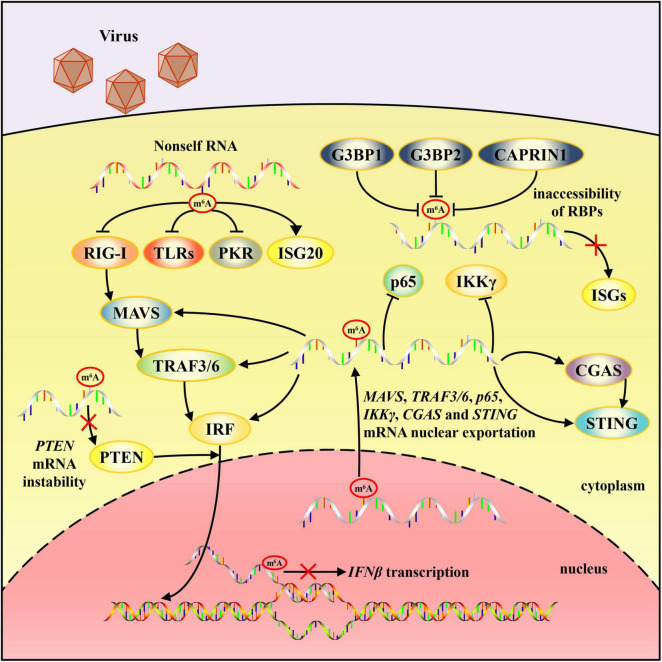
Schematic diagram of mechanisms by which m^6^A modification regulates non-self RNA recognition and innate immune responses. m^6^A modification in exogenous RNA prevent it from being identified by RNA sensors except for ISG20. Nuclear exportation of *MAVS*, *TRAF3/6*, *p65*, *IKK*γ, *CGAS*, and *STING* mRNA can be accelerated by m^6^A modification. m^6^A decoration may be an obstacle to IFNβ and ISGs expression. *PTEN*, *p65* and *IKK*γ mRNA instability can also be attributed to m^6^A modification.

**FIGURE 2:**
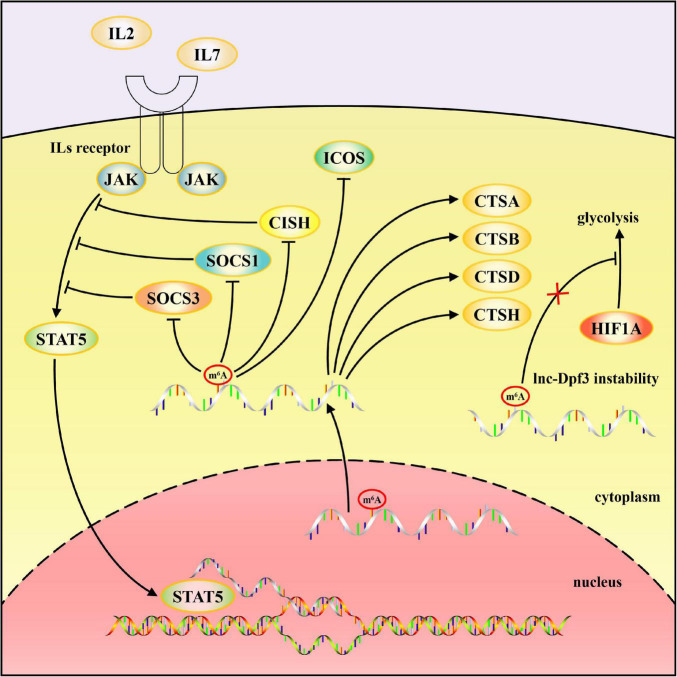
Schematic diagram of mechanisms by which m^6^A modification regulates adaptive immune responses. m^6^A modification of *SOCS1*, *SOCS3*, *CISH* mRNA, and *lnc-Dpf3* accelerate their decay. Translation of lysosomal cathepsins, including CTSA, CTSB, CTSD, and CTSH, are promoted by m^6^A and YTHDF1, whereas the expression of ICOS is inhibited by m^6^A modification.

The human immune system reacts to viral infection effectively by innate and adaptive immune responses. Many studies demonstrate that m^6^A modification regulates multiple steps of the antiviral immune response and plays an important role in the viral infection process (Figures 1, 2). In this review, we present up-to-date knowledge about m^6^A modification in regulating viral nucleic acid recognition, IFN production, and DC and macrophage maturation, among others. The involvement of m^6^A modification in antigen presentation, effector lymphocyte differentiation and other processes of adaptive immune response are also emphasized. These studies provide a basis in understanding the key role of m^6^A or other RNA modifications in infection and immunity in addition to providing new strategies for anti-infection immunotherapy development.

## Author Contributions

XC: conceptualization and supervision. YZ, HQ, and ZG: data curation. BZ and WW: writing—original draft. BZ, WW, and XC: writing—review and editing. BZ, WW, YZ, HQ, and ZG: visualization. All authors contributed to the article and approved the submitted version.

## Conflict of Interest

The authors declare that the research was conducted in the absence of any commercial or financial relationships that could be construed as a potential conflict of interest.

## Publisher’s Note

All claims expressed in this article are solely those of the authors and do not necessarily represent those of their affiliated organizations, or those of the publisher, the editors and the reviewers. Any product that may be evaluated in this article, or claim that may be made by its manufacturer, is not guaranteed or endorsed by the publisher.

## References

[B1] Alisjahbana A. Mohammad I. Gao Y. Evren E. Ringqvist E. Willinger T. (2020). Human macrophages and innate lymphoid cells: tissue-resident innate immunity in humanized mice. *Biochem. Pharmacol.* 174:113672. 10.1016/j.bcp.2019.113672 31634458

[B2] Anderson E. J. Rouphael N. G. Widge A. T. Jackson L. A. Roberts P. C. Makhene M. (2020). Safety and immunogenicity of SARS-CoV-2 mRNA-1273 vaccine in older adults. *N. Engl. J. Med.* 383 2427–2438. 10.1056/NEJMoa2028436 32991794 PMC7556339

[B3] Arguello A. E. DeLiberto A. N. Kleiner R. E. (2017). RNA chemical proteomics reveals the N(6)-Methyladenosine (m(6)A)-regulated protein-RNA interactome. *J. Am. Chem. Soc.* 139 17249–17252. 10.1021/jacs.7b09213 29140688

[B4] Bi Z. Liu Y. Zhao Y. Yao Y. Wu R. Liu Q. (2019). A dynamic reversible RNA N(6) -methyladenosine modification: current status and perspectives. *J. Cell Physiol.* 234 7948–7956. 10.1002/jcp.28014 30644095

[B5] Bidet K. Dadlani D. Garcia-Blanco M. A. (2014). G3BP1, G3BP2 and CAPRIN1 are required for translation of interferon stimulated mRNAs and are targeted by a dengue virus non-coding RNA. *PLoS Pathog.* 10:e1004242. 10.1371/journal.ppat.1004242 24992036 PMC4081823

[B6] Boccaletto P. Machnicka M. A. Purta E. Piatkowski P. Baginski B. Wirecki T. K. (2018). MODOMICS: a database of RNA modification pathways. 2017 update. *Nucleic Acids Res.* 46 D303–D307. 10.1093/nar/gkx1030 29106616 PMC5753262

[B7] Bou-Nader C. Gordon J. M. Henderson F. E. Zhang J. (2019). The search for a PKR code-differential regulation of protein kinase R activity by diverse RNA and protein regulators. *RNA* 25 539–556. 10.1261/rna.070169.118 30770398 PMC6467004

[B8] Bretou M. Saez P. J. Sanseau D. Maurin M. Lankar D. Chabaud M. (2017). Lysosome signaling controls the migration of dendritic cells. *Sci. Immunol.* 2:eaak9573. 10.1126/sciimmunol.aak9573 29079589

[B9] Cao X. (2020). COVID-19: immunopathology and its implications for therapy. *Nat. Rev. Immunol.* 20 269–270. 10.1038/s41577-020-0308-3 32273594 PMC7143200

[B10] Chen N. Xia P. Li S. Zhang T. Wang T. T. Zhu J. (2017). RNA sensors of the innate immune system and their detection of pathogens. *IUBMB Life* 69 297–304. 10.1002/iub.1625 28374903 PMC7165898

[B11] Chen S. Kumar S. Espada C. E. Tirumuru N. Cahill M. P. Hu L. (2021). N6-methyladenosine modification of HIV-1 RNA suppresses type-I interferon induction in differentiated monocytic cells and primary macrophages. *PLoS Pathog.* 17:e1009421. 10.1371/journal.ppat.1009421 33690734 PMC7984636

[B12] Chen Y. G. Chen R. Ahmad S. Verma R. Kasturi S. P. Amaya L. (2019). N6-Methyladenosine modification controls circular RNA immunity. *Mol. Cell* 76 96–109e109. 10.1016/j.molcel.2019.07.016 31474572 PMC6778039

[B13] Cohen J. (2020). Vaccine designers take first shots at COVID-19. *Science* 368 14–16. 10.1126/science.368.6486.14 32241928

[B14] Courtney D. G. Kennedy E. M. Dumm R. E. Bogerd H. P. Tsai K. Heaton N. S. (2017). Epitranscriptomic enhancement of influenza a virus gene expression and replication. *Cell Host Microbe* 22 377–386e375. 10.1016/j.chom.2017.08.004 28910636 PMC5615858

[B15] Dooling K. McClung N. Chamberland M. Marin M. Wallace M. Bell B. P. (2020). The advisory committee on immunization practices’ interim recommendation for allocating initial supplies of COVID-19 vaccine - United States, 2020. *MMWR Morb. Mortal. Wkly. Rep.* 69 1857–1859. 10.15585/mmwr.mm6949e1 33301429 PMC7737687

[B16] Durbin A. F. Wang C. Marcotrigiano J. Gehrke L. (2016). RNAs containing modified nucleotides fail to trigger RIG-I conformational changes for innate immune signaling. *mBio* 7 e833–e816. 10.1128/mBio.00833-16 * e00833-16, 27651356 PMC5030355

[B17] Edupuganti R. R. Geiger S. Lindeboom R. G. H. Shi H. Hsu P. J. Lu Z. (2017). N(6)-methyladenosine (m(6)A) recruits and repels proteins to regulate mRNA homeostasis. *Nat. Struct. Mol. Biol.* 24 870–878. 10.1038/nsmb.3462 28869609 PMC5725193

[B18] Feng Z. Li Q. Meng R. Yi B. Xu Q. (2018). METTL3 regulates alternative splicing of MyD88 upon the lipopolysaccharide-induced inflammatory response in human dental pulp cells. *J. Cell Mol. Med.* 22 2558–2568. 10.1111/jcmm.13491 29502358 PMC5908103

[B19] Fu Y. Dominissini D. Rechavi G. He C. (2014). Gene expression regulation mediated through reversible m(6)A RNA methylation. *Nat. Rev. Genet.* 15 293–306. 10.1038/nrg3724 24662220

[B20] Fu Y. Zorman B. Sumazin P. Sanna P. P. Repunte-Canonigo V. (2019). Epitranscriptomics: correlation of N6-methyladenosine RNA methylation and pathway dysregulation in the hippocampus of HIV transgenic rats. *PLoS One* 14:e0203566. 10.1371/journal.pone.0203566 30653517 PMC6336335

[B21] Ge Y. Ling T. Wang Y. Jia X. Xie X. Chen R. (2021). Degradation of WTAP blocks antiviral responses by reducing the m(6) a levels of IRF3 and IFNAR1 mRNA. *EMBO Rep.* 22:e52101. 10.15252/embr.202052101 34467630 PMC8567250

[B22] Gokhale N. S. McIntyre A. B. R. Mattocks M. D. Holley C. L. Lazear H. M. Mason C. E. (2020). Altered m(6)a modification of specific cellular transcripts affects flaviviridae infection. *Mol Cell* 77 542–555e548. 10.1016/j.molcel.2019.11.007 31810760 PMC7007864

[B23] Grozhik A. V. Linder B. Olarerin-George A. O. Jaffrey S. R. (2017). Mapping m(6)A at individual-nucleotide resolution using crosslinking and immunoprecipitation (miCLIP). *Methods Mol. Biol.* 1562 55–78. 10.1007/978-1-4939-6807-7_5 28349454 PMC5562447

[B24] Gu X. Zhang Y. Li D. Cai H. Cai L. Xu Q. (2020). N6-methyladenosine demethylase FTO promotes M1 and M2 macrophage activation. *Cell Signal* 69:109553. 10.1016/j.cellsig.2020.109553 32018056

[B25] Han D. Liu J. Chen C. Dong L. Liu Y. Chang R. (2019). Anti-tumour immunity controlled through mRNA m(6)A methylation and YTHDF1 in dendritic cells. *Nature* 566 270–274. 10.1038/s41586-019-0916-x 30728504 PMC6522227

[B26] Hao H. Hao S. Chen H. Chen Z. Zhang Y. Wang J. (2019). N6-methyladenosine modification and METTL3 modulate enterovirus 71 replication. *Nucleic Acids Res.* 47 362–374. 10.1093/nar/gky1007 30364964 PMC6326802

[B27] Hesser C. R. Karijolich J. Dominissini D. He C. Glaunsinger B. A. (2018). N6-methyladenosine modification and the YTHDF2 reader protein play cell type specific roles in lytic viral gene expression during Kaposi’s sarcoma-associated herpesvirus infection. *PLoS Pathog.* 14:e1006995. 10.1371/journal.ppat.1006995 29659627 PMC5919695

[B28] Hou J. Zhang H. Liu J. Zhao Z. Wang J. Lu Z. (2019). YTHDF2 reduction fuels inflammation and vascular abnormalization in hepatocellular carcinoma. *Mol. Cancer* 18:163. 10.1186/s12943-019-1082-3 31735169 PMC6859620

[B29] Imaeda A. Tomoike F. Hayakawa M. Nakamoto K. Kimura Y. Abe N. (2019). N(6)-methyl adenosine in siRNA evades immune response without reducing RNAi activity. *Nucleosides Nucleotides Nucleic Acids* 38 972–979. 10.1080/15257770.2019.1641205 31298608

[B30] Imam H. Khan M. Gokhale N. S. McIntyre A. B. R. Kim G. W. Jang J. Y. (2018). N6-methyladenosine modification of hepatitis B virus RNA differentially regulates the viral life cycle. *Proc. Natl. Acad. Sci. U.S.A.* 115 8829–8834. 10.1073/pnas.1808319115 30104368 PMC6126736

[B31] Imam H. Kim G. W. Mir S. A. Khan M. Siddiqui A. (2020). Interferon-stimulated gene 20 (ISG20) selectively degrades N6-methyladenosine modified Hepatitis B Virus transcripts. *PLoS Pathog.* 16:e1008338. 10.1371/journal.ppat.1008338 32059034 PMC7046284

[B32] Jurczyszak D. Zhang W. Terry S. N. Kehrer T. Bermudez Gonzalez M. C. McGregor E. (2020). HIV protease cleaves the antiviral m6A reader protein YTHDF3 in the viral particle. *PLoS Pathog.* 16:e1008305. 10.1371/journal.ppat.1008305 32053707 PMC7043784

[B33] Kariko K. Buckstein M. Ni H. Weissman D. (2005). Suppression of RNA recognition by Toll-like receptors: the impact of nucleoside modification and the evolutionary origin of RNA. *Immunity* 23 165–175. 10.1016/j.immuni.2005.06.008 16111635

[B34] Kastan J. P. Tremblay M. W. Brown M. C. Trimarco J. D. Dobrikova E. Y. Dobrikov M. I. (2021). Enterovirus 2A(pro) cleavage of the YTHDF m(6)a readers implicates YTHDF3 as a mediator of Type I interferon-driven JAK/STAT signaling. *mBio* 12 e116–e121. 10.1128/mBio.00116-21 33849973 PMC8092205

[B35] Kennedy E. M. Bogerd H. P. Kornepati A. V. Kang D. Ghoshal D. Marshall J. B. (2016). Posttranscriptional m(6)a editing of HIV-1 mRNAs enhances viral gene expression. *Cell Host Microbe* 19 675–685. 10.1016/j.chom.2016.04.002 27117054 PMC4867121

[B36] Kennedy E. M. Courtney D. G. Tsai K. Cullen B. R. (2017). Viral epitranscriptomics. *J. Virol.* 91 e2263–e2216. 10.1128/JVI.02263-16 28250115 PMC5391447

[B37] Kim G. W. Imam H. Khan M. Mir S. A. Kim S. J. Yoon S. K. (2020a). HBV-induced increased N6 methyladenosine modification of PTEN RNA affects innate immunity and contributes to HCC. *Hepatology* 73 533–547. 10.1002/hep.31313 32394474 PMC7655655

[B38] Kim G. W. Imam H. Khan M. Siddiqui A. (2020b). N (6)-Methyladenosine modification of hepatitis B and C viral RNAs attenuates host innate immunity *via* RIG-I signaling. *J. Biol. Chem.* 295 13123–13133. 10.1074/jbc.RA120.014260 32719095 PMC7489896

[B39] Kim G. W. Siddiqui A. (2021). Hepatitis B virus X protein recruits methyltransferases to affect cotranscriptional N6-methyladenosine modification of viral/host RNAs. *Proc. Natl. Acad. Sci. U.S.A.* 118 e2019455118. 10.1073/pnas.2019455118 33397803 PMC7826408

[B40] Lang F. Singh R. K. Pei Y. Zhang S. Sun K. Robertson E. S. (2019). EBV epitranscriptome reprogramming by METTL14 is critical for viral-associated tumorigenesis. *PLoS Pathog.* 15:e1007796. 10.1371/journal.ppat.1007796 31226160 PMC6588254

[B41] Li H. B. Tong J. Zhu S. Batista P. J. Duffy E. E. Zhao J. (2017). m(6)A mRNA methylation controls T cell homeostasis by targeting the IL-7/STAT5/SOCS pathways. *Nature* 548 338–342. 10.1038/nature23450 28792938 PMC5729908

[B42] Li M. M. MacDonald M. R. Rice C. M. (2015). To translate, or not to translate: viral and host mRNA regulation by interferon-stimulated genes. *Trends Cell. Biol.* 25 320–329. 10.1016/j.tcb.2015.02.001 25748385 PMC4441850

[B43] Li N. Hui H. Bray B. Gonzalez G. M. Zeller M. Anderson K. G. (2021). METTL3 regulates viral m6A RNA modification and host cell innate immune responses during SARS-CoV-2 infection. *Cell Rep.* 35:109091. 10.1016/j.celrep.2021.109091 33961823 PMC8090989

[B44] Liao Y. Tong L. Tang L. Wu S. (2017). The role of cold-inducible RNA binding protein in cell stress response. *Int. J. Cancer* 141 2164–2173. 10.1002/ijc.30833 28608439

[B45] Lichinchi G. Gao S. Saletore Y. Gonzalez G. M. Bansal V. Wang Y. (2016a). Dynamics of the human and viral m(6)A RNA methylomes during HIV-1 infection of T cells. *Nat. Microbiol.* 1:16011. 10.1038/nmicrobiol.2016.11 27572442 PMC6053355

[B46] Lichinchi G. Zhao B. S. Wu Y. Lu Z. Qin Y. He C. (2016b). Dynamics of human and viral RNA methylation during Zika virus infection. *Cell Host Microbe* 20 666–673. 10.1016/j.chom.2016.10.002 27773536 PMC5155635

[B47] Liu N. Zhou K. I. Parisien M. Dai Q. Diatchenko L. Pan T. (2017). N6-methyladenosine alters RNA structure to regulate binding of a low-complexity protein. *Nucleic Acids Res.* 45 6051–6063. 10.1093/nar/gkx141 28334903 PMC5449601

[B48] Liu J. Zhang X. Chen K. Cheng Y. Liu S. Xia M. (2019a). CCR7 chemokine receptor-inducible lnc-Dpf3 restrains dendritic cell migration by inhibiting HIF-1alpha-mediated glycolysis. *Immunity* 50 600–615 e615. 10.1016/j.immuni.2019.01.021 30824325

[B49] Liu Q. Li M. Jiang L. Jiang R. Fu B. (2019b). METTL3 promotes experimental osteoarthritis development by regulating inflammatory response and apoptosis in chondrocyte. *Biochem. Biophys. Res. Commun.* 516 22–27. 10.1016/j.bbrc.2019.05.168 31186141

[B50] Liu Y. Liu Z. Tang H. Shen Y. Gong Z. Xie N. (2019c). The N(6)-methyladenosine (m(6)A)-forming enzyme METTL3 facilitates M1 macrophage polarization through the methylation of STAT1 mRNA. *Am. J. Physiol. Cell Physiol.* 317 C762–C775. 10.1152/ajpcell.00212.2019 31365297

[B51] Liu Y. You Y. Lu Z. Yang J. Li P. Liu L. (2019d). N (6)-methyladenosine RNA modification-mediated cellular metabolism rewiring inhibits viral replication. *Science* 365 1171–1176. 10.1126/science.aax4468 31439758

[B52] Lu M. Xue M. Wang H. T. Kairis E. L. Ahmad S. Wei J. (2021). Nonsegmented negative-sense RNA viruses utilize N (6)-Methyladenosine (m(6)A) as a common strategy to evade host innate immunity. *J. Virol.* 95 e1939–e1920. 10.1128/JVI.01939-20 33536170 PMC8104112

[B53] Lu M. Zhang Z. Xue M. Zhao B. S. Harder O. Li A. (2020). N(6)-methyladenosine modification enables viral RNA to escape recognition by RNA sensor RIG-I. *Nat. Microbiol.* 5 584–598. 10.1038/s41564-019-0653-9 32015498 PMC7137398

[B54] Malik G. Zhou Y. (2020). Innate immune sensing of influenza a virus. *Viruses* 12:755. 10.3390/v12070755 32674269 PMC7411791

[B55] McFadden M. J. Gokhale N. S. Horner S. M. (2017). Protect this house: cytosolic sensing of viruses. *Curr. Opin. Virol.* 22 36–43. 10.1016/j.coviro.2016.11.012 27951430 PMC5346041

[B56] McFadden M. J. McIntyre A. B. R. Mourelatos H. Abell N. S. Gokhale N. S. Ipas H. (2021). Post-transcriptional regulation of antiviral gene expression by N6-methyladenosine. *Cell Rep.* 34:108798. 10.1016/j.celrep.2021.108798 33657363 PMC7981787

[B57] Nachtergaele S. He C. (2018). Chemical modifications in the life of an mRNA transcript. *Annu. Rev. Genet.* 52 349–372. 10.1146/annurev-genet-120417-031522 30230927 PMC6436393

[B58] Oshiumi H. Kouwaki T. Seya T. (2016). Accessory factors of cytoplasmic viral RNA sensors required for antiviral innate immune response. *Front. Immunol.* 7:200. 10.3389/fimmu.2016.00200 27252702 PMC4879126

[B59] Ovcharenko A. Rentmeister A. (2018). Emerging approaches for detection of methylation sites in RNA. *Open Biol.* 8:180121. 10.1098/rsob.180121 30185602 PMC6170510

[B60] Pardi N. Parkhouse K. Kirkpatrick E. McMahon M. Zost S. J. Mui B. L. (2018). Nucleoside-modified mRNA immunization elicits influenza virus hemagglutinin stalk-specific antibodies. *Nat. Commun.* 9:3361. 10.1038/s41467-018-05482-0 30135514 PMC6105651

[B61] Pardi N. Secreto A. J. Shan X. Debonera F. Glover J. Yi Y. (2017). Administration of nucleoside-modified mRNA encoding broadly neutralizing antibody protects humanized mice from HIV-1 challenge. *Nat. Commun.* 8:14630. 10.1038/ncomms14630 28251988 PMC5337964

[B62] Qiu W. Zhang Q. Zhang R. Lu Y. Wang X. Tian H. (2021). N(6)-methyladenosine RNA modification suppresses antiviral innate sensing pathways *via* reshaping double-stranded RNA. *Nat. Commun.* 12:1582. 10.1038/s41467-021-21904-y 33707441 PMC7952553

[B63] Rakebrandt N. Littringer K. Joller N. (2016). Regulatory T cells: balancing protection versus pathology. *Swiss Med. Wkly.* 146:w14343. 10.4414/smw.2016.14343 27497235

[B64] Richner J. M. Himansu S. Dowd K. A. Butler S. L. Salazar V. Fox J. M. (2017). Modified mRNA vaccines protect against zika virus infection. *Cell* 168 1114–1125 e1110. 10.1016/j.cell.2017.02.017 28222903 PMC5388441

[B65] Ringeard M. Marchand V. Decroly E. Motorin Y. Bennasser Y. (2019). FTSJ3 is an RNA 2’-O-methyltransferase recruited by HIV to avoid innate immune sensing. *Nature* 565 500–504. 10.1038/s41586-018-0841-4 30626973

[B66] Rubio R. M. Depledge D. P. Bianco C. Thompson L. Mohr I. (2018). RNA m(6) a modification enzymes shape innate responses to DNA by regulating interferon beta. *Genes Dev.* 32 1472–1484. 10.1101/gad.319475.118 30463905 PMC6295168

[B67] Schlee M. Hartmann G. (2016). Discriminating self from non-self in nucleic acid sensing. *Nat. Rev. Immunol.* 16 566–580. 10.1038/nri.2016.78 27455396 PMC7097691

[B68] Shi H. Wei J. He C. (2019). Where, when, and how: context-dependent functions of RNA methylation writers, readers, and erasers. *Mol. Cell* 74 640–650. 10.1016/j.molcel.2019.04.025 31100245 PMC6527355

[B69] Sitaraman R. (2016). The role of DNA restriction-modification systems in the biology of *Bacillus anthracis*. *Front. Microbiol.* 7:11. 10.3389/fmicb.2016.00011 26834729 PMC4722110

[B70] Tan B. Liu H. Zhang S. da Silva S. R. Zhang L. Meng J. (2018). Viral and cellular N(6)-methyladenosine and N(6),2’-O-dimethyladenosine epitranscriptomes in the KSHV life cycle. *Nat. Microbiol.* 3 108–120. 10.1038/s41564-017-0056-8 29109479 PMC6138870

[B71] Tirumuru N. Wu L. (2019). HIV-1 envelope proteins up-regulate N (6)-methyladenosine levels of cellular RNA independently of viral replication. *J. Biol. Chem.* 294 3249–3260. 10.1074/jbc.RA118.005608 30617182 PMC6398121

[B72] Tirumuru N. Zhao B. S. Lu W. Lu Z. He C. Wu L. (2016). N(6)-methyladenosine of HIV-1 RNA regulates viral infection and HIV-1 Gag protein expression. *Elife* 5:e15528. 10.7554/eLife.15528 27371828 PMC4961459

[B73] Tong J. Cao G. Zhang T. Sefik E. Amezcua Vesely M. C. Broughton J. P. (2018). m(6)A mRNA methylation sustains Treg suppressive functions. *Cell Res.* 28 253–256. 10.1038/cr.2018.7 29303144 PMC5799823

[B74] Wang H. Hu X. Huang M. Liu J. Gu Y. Ma L. (2019). Mettl3-mediated mRNA m(6)A methylation promotes dendritic cell activation. *Nat. Commun.* 10:1898. 10.1038/s41467-019-09903-6 31015515 PMC6478715

[B75] Wang J. Yan S. Lu H. Wang S. Xu D. (2019). METTL3 attenuates LPS-induced inflammatory response in macrophages *via* NF-kappaB signaling pathway. *Mediators Inflamm.* 2019:3120391. 10.1155/2019/3120391 31772500 PMC6854952

[B76] Wang L. Wen M. Cao X. (2019). Nuclear hnRNPA2B1 initiates and amplifies the innate immune response to DNA viruses. *Science* 365:eaav0758. 10.1126/science.aav0758 31320558

[B77] Wang X. Zhao B. S. Roundtree I. A. Lu Z. Han D. Ma H. (2015). N(6)-methyladenosine modulates messenger RNA translation efficiency. *Cell* 161 1388–1399. 10.1016/j.cell.2015.05.014 26046440 PMC4825696

[B78] Winkler R. Gillis E. Lasman L. Safra M. Geula S. Soyris C. (2019). m(6)A modification controls the innate immune response to infection by targeting type I interferons. *Nat. Immunol.* 20 173–182. 10.1038/s41590-018-0275-z 30559377

[B79] Wu F. Cheng W. Zhao F. Tang M. Diao Y. Xu R. (2019). Association of N6-methyladenosine with viruses and related diseases. *Virol. J.* 16:133. 10.1186/s12985-019-1236-3 31711514 PMC6849232

[B80] Xu J. Cai Y. Ma Z. Jiang B. Liu W. Cheng J. (2021). The RNA helicase DDX5 promotes viral infection *via* regulating N6-methyladenosine levels on the DHX58 and NFkappaB transcripts to dampen antiviral innate immunity. *PLoS Pathog.* 17:e1009530. 10.1371/journal.ppat.1009530 33909701 PMC8081163

[B81] Xue M. Zhao B. S. Zhang Z. Lu M. Harder O. Chen P. (2019). Viral N(6)-methyladenosine upregulates replication and pathogenesis of human respiratory syncytial virus. *Nat. Commun.* 10:4595. 10.1038/s41467-019-12504-y 31597913 PMC6785563

[B82] Yang J. Wang H. Zhang W. (2019). Regulation of virus replication and T cell homeostasis by N(6)-Methyladenosine. *Virol. Sin.* 34 22–29. 10.1007/s12250-018-0075-5 30671921 PMC6420589

[B83] Yao M. Dong Y. Wang Y. Liu H. Ma H. Zhang H. (2020). N(6)-methyladenosine modifications enhance enterovirus 71 ORF translation through METTL3 cytoplasmic distribution. *Biochem. Biophys. Res. Commun.* 527 297–304. 10.1016/j.bbrc.2020.04.088 32446384

[B84] Yasmin R. Siraj S. Hassan A. Khan A. R. Abbasi R. Ahmad N. (2015). Epigenetic regulation of inflammatory cytokines and associated genes in human malignancies. *Mediators Inflamm.* 2015:201703. 10.1155/2015/201703 25814785 PMC4359879

[B85] Yu R. Li Q. Feng Z. Cai L. Xu Q. (2019). m6A Reader YTHDF2 Regulates LPS-induced inflammatory response. *Int. J. Mol. Sci.* 20:1323. 10.3390/ijms20061323 30875984 PMC6470741

[B86] Yuen K. C. Xu B. Krantz I. D. Gerton J. L. (2016). NIPBL controls RNA biogenesis to prevent activation of the stress kinase PKR. *Cell Rep.* 14 93–102. 10.1016/j.celrep.2015.12.012 26725122 PMC4904785

[B87] Zaccara S. Ries R. J. Jaffrey S. R. (2019). Reading, writing and erasing mRNA methylation. *Nat. Rev. Mol. Cell Biol.* 20 608–624. 10.1038/s41580-019-0168-5 31520073

[B88] Zhang Y. Wang X. Zhang X. Wang J. Ma Y. Zhang L. (2019). RNA-binding protein YTHDF3 suppresses interferon-dependent antiviral responses by promoting FOXO3 translation. *Proc. Natl. Acad. Sci. U.S.A.* 116 976–981. 10.1073/pnas.1812536116 30591559 PMC6338863

[B89] Zheng Q. Hou J. Zhou Y. Li Z. Cao X. (2017). The RNA helicase DDX46 inhibits innate immunity by entrapping m(6)A-demethylated antiviral transcripts in the nucleus. *Nat. Immunol.* 18 1094–1103. 10.1038/ni.3830 28846086

[B90] Zhu J. (2018). T helper cell differentiation, heterogeneity, and plasticity. *Cold Spring Harb. Perspect. Biol.* 10 a030338. 10.1101/cshperspect.a030338 28847903 PMC6169815

[B91] Zhu Y. Zhao Y. Zou L. Zhang D. Aki D. Liu Y. C. (2019). The E3 ligase VHL promotes follicular helper T cell differentiation *via* glycolytic-epigenetic control. *J. Exp. Med.* 216 1664–1681. 10.1084/jem.20190337 31123085 PMC6605754

